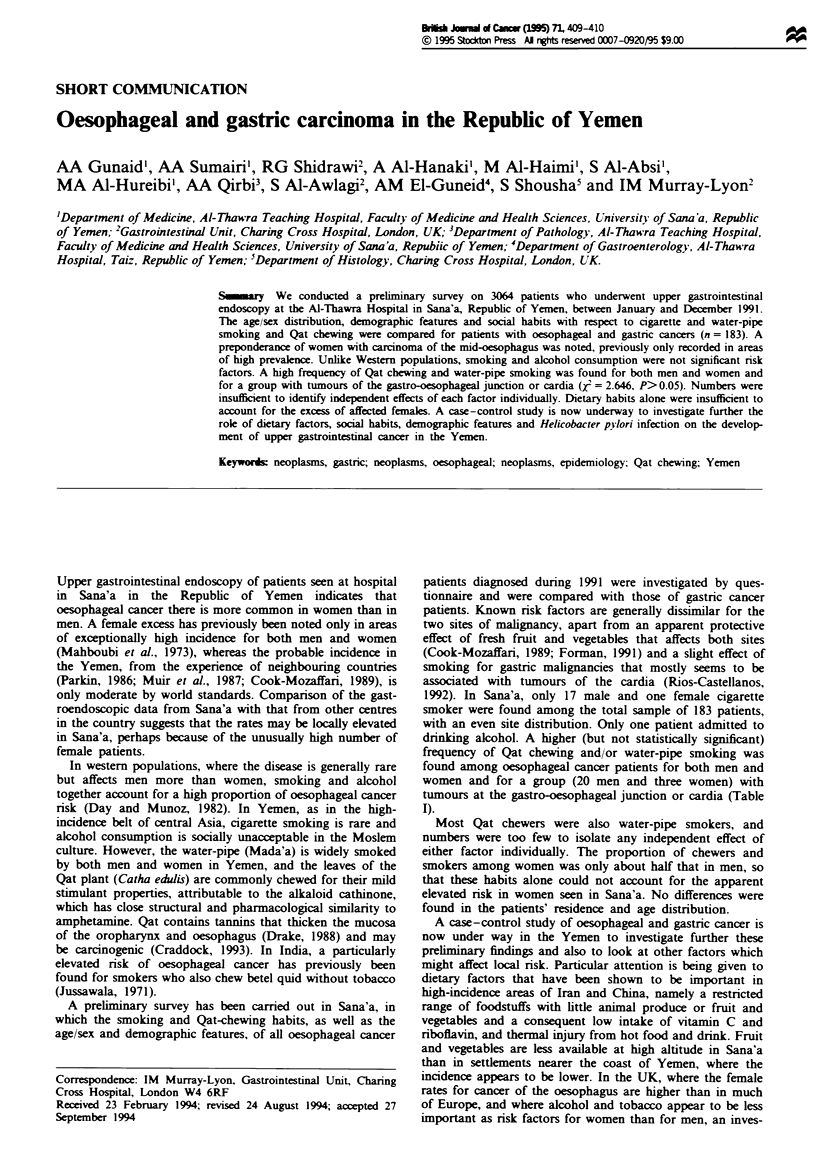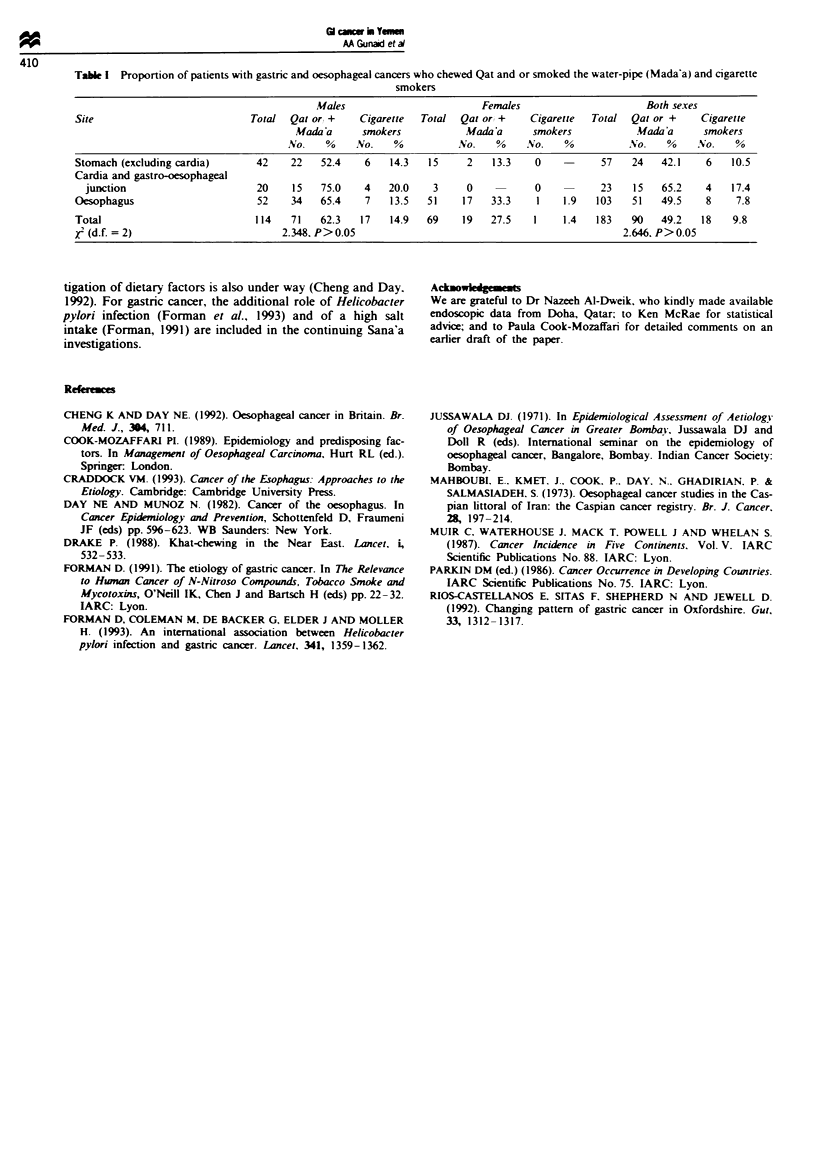# Oesophageal and gastric carcinoma in the Republic of Yemen.

**DOI:** 10.1038/bjc.1995.83

**Published:** 1995-02

**Authors:** A. A. Gunaid, A. A. Sumairi, R. G. Shidrawi, A. al-Hanaki, M. al-Haimi, S. al-Absi, M. A. al-Hureibi, A. A. Qirbi, S. al-Awlagi, A. M. el-Guneid

**Affiliations:** Department of Medicine, Al-Thawra Teaching Hospital, Faculty of Medicine and Health Sciences, University of Sana'a, Republic of Yemen.

## Abstract

We conducted a preliminary survey on 3064 patients who underwent upper gastrointestinal endoscopy at the Al-Thawra Hospital in Sana'a, Republic of Yemen, between January and December 1991. The age/sex distribution, demographic features and social habits with respect to cigarette and water-pipe smoking and Qat chewing were compared for patients with oesophageal and gastric cancers (n = 183). A preponderance of women with carcinoma of the mid-oesophageal was noted, previously only recorded in areas of high prevalence. Unlike Western populations, smoking and alcohol consumption were not significant risk factors. A high frequency of Qat chewing and water-pipe smoking was found for both men and women and for a group with tumours of the gastro-oesophageal junction or cardia (chi 2 = 2.646, P > 0.05). Numbers were insufficient to identify independent effects of each factor individually. Dietary habits alone were insufficient to account for the excess of affected females. A case-control study is now underway to investigate further the role of dietary factors, social habits, demographic features and Helicobacter pylori infection on the development of upper gastrointestinal cancer in the Yemen.


© 1995 Stockton Press All rights reserved 0007-0920/95 $9.00

SHORT COMMUNICATION

### Oesophageal and gastric carcinoma in the Republic of Yemen

AA Gunaid[1], AA Sumairi[1], RG Shidrawi[2], A Al-Hanaki[1], M Al-Haimi[1], S Al-Absi[1], MA Al-Hureibi[1], AA Qirbi[3], S Al-Awlagi[2], AM El-Guneid[4], S Shousha[5] and IM Murray-Lyon[2]

[1]*Department of Medicine, Al-Thawra Teaching Hospital, Faculty of Medicine and Health Sciences, University of Sana'a, Republic of Yemen;* [2]*Gastrointestinal Unit, Charing Cross Hospital, London, UK;* [3]*Department of Pathology, Al-Thawra Teaching Hospital, Faculty of Medicine and Health Sciences, University of Sana'a, Repubiic of Yemen;* [4]*Department of Gastroenterology, Al-Thawra Hospital, Taiz, Republic of Yemen;* [5]*Department of Histology, Charing Cross Hospital, London, UK.*

**Summary** We conducted a preliminary survey on 3064 patients who underwent upper gastrointestinal endoscopy at the Al-Thawra Hospital in Sana'a, Republic of Yemen, between January and December 1991. The age/sex distribution, demographic features and social habits with respect to cigarette and water-pipe smoking and Qat chewing were compared for patients with oesophageal and gastric cancers ($n = 183$). A preponderance of women with carcinoma of the mid-oesophagus was noted, previously only recorded in areas of high prevalence. Unlike Western populations, smoking and alcohol consumption were not significant risk factors. A high frequency of Qat chewing and water-pipe smoking was found for both men and women and for a group with tumours of the gastro-oesophageal junction or cardia ($\chi^2 = 2.646$, $P > 0.05$). Numbers were insufficient to identify independent effects of each factor individually. Dietary habits alone were insufficient to account for the excess of affected females. A case-control study is now underway to investigate further the role of dietary factors, social habits, demographic features and *Helicobacter pylori* infection on the development of upper gastrointestinal cancer in the Yemen.

**Keywords:** neoplasms, gastric; neoplasms, oesophageal; neoplasms, epidemiology; Qat chewing; Yemen

Upper gastrointestinal endoscopy of patients seen at hospital in Sana'a in the Republic of Yemen indicates that oesophageal cancer there is more common in women than in men. A female excess has previously been noted only in areas of exceptionally high incidence for both men and women (Mahboubi *et al.*, 1973), whereas the probable incidence in the Yemen, from the experience of neighbouring countries (Parkin, 1986; Muir *et al.*, 1987; Cook-Mozaffari, 1989), is only moderate by world standards. Comparison of the gastroendoscopic data from Sana'a with that from other centres in the country suggests that the rates may be locally elevated in Sana'a, perhaps because of the unusually high number of female patients.

In western populations, where the disease is generally rare but affects men more than women, smoking and alcohol together account for a high proportion of oesophageal cancer risk (Day and Munoz, 1982). In Yemen, as in the high-incidence belt of central Asia, cigarette smoking is rare and alcohol consumption is socially unacceptable in the Moslem culture. However, the water-pipe (Mada'a) is widely smoked by both men and women in Yemen, and the leaves of the Qat plant (*Catha edulis*) are commonly chewed for their mild stimulant properties, attributable to the alkaloid cathinone, which has close structural and pharmacological similarity to amphetamine. Qat contains tannins that thicken the mucosa of the oropharynx and oesophagus (Drake, 1988) and may be carcinogenic (Craddock, 1993). In India, a particularly elevated risk of oesophageal cancer has previously been found for smokers who also chew betel quid without tobacco (Jussawala, 1971).

A preliminary survey has been carried out in Sana'a, in which the smoking and Qat-chewing habits, as well as the age/sex and demographic features, of all oesophageal cancer patients diagnosed during 1991 were investigated by questionnaire and were compared with those of gastric cancer patients. Known risk factors are generally dissimilar for the two sites of malignancy, apart from an apparent protective effect of fresh fruit and vegetables that affects both sites (Cook-Mozaffari, 1989; Forman, 1991) and a slight effect of smoking for gastric malignancies that mostly seems to be associated with tumours of the cardia (Rios-Castellanos, 1992). In Sana'a, only 17 male and one female cigarette smoker were found among the total sample of 183 patients, with an even site distribution. Only one patient admitted to drinking alcohol. A higher (but not statistically significant) frequency of Qat chewing and/or water-pipe smoking was found among oesophageal cancer patients for both men and women and for a group (20 men and three women) with tumours at the gastro-oesophageal junction or cardia (Table I).

Most Qat chewers were also water-pipe smokers, and numbers were too few to isolate any independent effect of either factor individually. The proportion of chewers and smokers among women was only about half that in men, so that these habits alone could not account for the apparent elevated risk in women seen in Sana'a. No differences were found in the patients' residence and age distribution.

A case-control study of oesophageal and gastric cancer is now under way in the Yemen to investigate further these preliminary findings and also to look at other factors which might affect local risk. Particular attention is being given to dietary factors that have been shown to be important in high-incidence areas of Iran and China, namely a restricted range of foodstuffs with little animal produce or fruit and vegetables and a consequent low intake of vitamin C and riboflavin, and thermal injury from hot food and drink. Fruit and vegetables are less available at high altitude in Sana'a than in settlements nearer the coast of Yemen, where the incidence appears to be lower. In the UK, where the female rates for cancer of the oesophagus are higher than in much of Europe, and where alcohol and tobacco appear to be less important as risk factors for women than for men, an inves-

Correspondence: IM Murray-Lyon, Gastrointestinal Unit, Charing Cross Hospital, London W4 6RF
Received 23 February 1994; revised 24 August 1994; accepted 27 September 1994

**Table I** Proportion of patients with gastric and oesophageal cancers who chewed Qat and or smoked the water-pipe (Mada'a) and cigarette smokers

| Site | Males Total | Males Qat or + Mada'a No. | Males Qat or + Mada'a % | Males Cigarette smokers No. | Males Cigarette smokers % | Females Total | Females Qat or + Mada'a No. | Females Qat or + Mada'a % | Females Cigarette smokers No. | Females Cigarette smokers % | Both sexes Total | Both sexes Qat or + Mada'a No. | Both sexes Qat or + Mada'a % | Both sexes Cigarette smokers No. | Both sexes Cigarette smokers % |
|---|---|---|---|---|---|---|---|---|---|---|---|---|---|---|---|
| Stomach (excluding cardia) | 42 | 22 | 52.4 | 6 | 14.3 | 15 | 2 | 13.3 | 0 | — | 57 | 24 | 42.1 | 6 | 10.5 |
| Cardia and gastro-oesophageal junction | 20 | 15 | 75.0 | 4 | 20.0 | 3 | 0 | — | 0 | — | 23 | 15 | 65.2 | 4 | 17.4 |
| Oesophagus | 52 | 34 | 65.4 | 7 | 13.5 | 51 | 17 | 33.3 | 1 | 1.9 | 103 | 51 | 49.5 | 8 | 7.8 |
| Total | 114 | 71 | 62.3 | 17 | 14.9 | 69 | 19 | 27.5 | 1 | 1.4 | 183 | 90 | 49.2 | 18 | 9.8 |
| $\chi^2$ (d.f. = 2) | | 2.348, $P>0.05$ | | | | | | | | | | 2.646, $P>0.05$ | | | |

tigation of dietary factors is also under way (Cheng and Day, 1992). For gastric cancer, the additional role of *Helicobacter pylori* infection (Forman *et al.*, 1993) and of a high salt intake (Forman, 1991) are included in the continuing Sana'a investigations.

**Acknowledgements**

We are grateful to Dr Nazeeh Al-Dweik, who kindly made available endoscopic data from Doha, Qatar; to Ken McRae for statistical advice; and to Paula Cook-Mozaffari for detailed comments on an earlier draft of the paper.

**References**

CHENG K AND DAY NE. (1992). Oesophageal cancer in Britain. *Br. Med. J.*, **304**, 711.

COOK-MOZAFFARI PI. (1989). Epidemiology and predisposing factors. In *Management of Oesophageal Carcinoma*, Hurt RL (ed.). Springer: London.

CRADDOCK VM. (1993). *Cancer of the Esophagus: Approaches to the Etiology*. Cambridge: Cambridge University Press.

DAY NE AND MUNOZ N. (1982). Cancer of the oesophagus. In *Cancer Epidemiology and Prevention*, Schottenfeld D, Fraumeni JF (eds) pp. 596-623. WB Saunders: New York.

DRAKE P. (1988). Khat-chewing in the Near East. *Lancet*, **i**, 532-533.

FORMAN D. (1991). The etiology of gastric cancer. In *The Relevance to Human Cancer of N-Nitroso Compounds, Tobacco Smoke and Mycotoxins*, O'Neill IK, Chen J and Bartsch H (eds) pp. 22-32. IARC: Lyon.

FORMAN D, COLEMAN M, DE BACKER G, ELDER J AND MOLLER H. (1993). An international association between *Helicobacter pylori* infection and gastric cancer. *Lancet*, **341**, 1359-1362.

JUSSAWALA DJ. (1971). In *Epidemiological Assessment of Aetiology of Oesophageal Cancer in Greater Bombay*, Jussawala DJ and Doll R (eds). International seminar on the epidemiology of oesophageal cancer, Bangalore, Bombay. Indian Cancer Society: Bombay.

MAHBOUBI, E., KMET, J., COOK, P., DAY, N., GHADIRIAN, P. & SALMASIADEH, S. (1973). Oesophageal cancer studies in the Caspian littoral of Iran: the Caspian cancer registry. *Br. J. Cancer*, **28**, 197-214.

MUIR C, WATERHOUSE J, MACK T, POWELL J AND WHELAN S. (1987). *Cancer Incidence in Five Continents*, Vol. V. IARC Scientific Publications No. 88. IARC: Lyon.

PARKIN DM (ed.) (1986). *Cancer Occurrence in Developing Countries*. IARC Scientific Publications No. 75. IARC: Lyon.

RIOS-CASTELLANOS E, SITAS F, SHEPHERD N AND JEWELL D. (1992). Changing pattern of gastric cancer in Oxfordshire. *Gut*, **33**, 1312-1317.